# Genetic Insights Into the Relationship Between Menstrual Factors and Site‐/Age‐Specific Bone Mineral Density

**DOI:** 10.1155/ijog/9979878

**Published:** 2025-11-10

**Authors:** Sheng Li, Yan-Yu Zhu, Xiao Hu, Qian-Qian Shi, Jia-Jun Deng, Hai-Fen Wei, Chun-Rong Ma, Hai-Feng Pan, Peng Wang

**Affiliations:** ^1^ Department of Health Promotion and Behavioral Sciences, School of Public Health, Anhui Medical University, Hefei, Anhui, China, ahmu.edu.cn; ^2^ Institute of Kidney Disease, Inflammation & Immunity Mediated Diseases, The Second Hospital of Anhui Medical University, Hefei, Anhui, China, ahmu.edu.cn; ^3^ Department of Epidemiology and Biostatistics, School of Public Health, Anhui Medical University, Hefei, Anhui, China, ahmu.edu.cn; ^4^ Tongan Street Community Health Center, Hefei, Anhui, China

**Keywords:** bone mineral density, causality, menstrual factors, osteoporosis

## Abstract

**Backgrounds:**

Observational studies have suggested possible links between reproductive factors and bone mineral density (BMD) and osteoporosis (OP), but the causal impact of menstrual factors on BMD remains obscure.

**Objectives:**

This study is aimed at inferring causal associations between key menstrual factors and site‐/age‐specific BMD.

**Methods:**

Genetic variants associated with age at menarche (AAM), last menstrual period (LMP), and length of menstrual cycle (LMC) were obtained from genome‐wide association studies (GWASs). The site‐/age‐specific BMD genetic data were derived from the Genetic Factors for OP Consortium (GEFOS). Causal associations were evaluated using a two‐sample Mendelian randomization (MR) approach and a multivariable MR (MVMR) model.

**Results:**

LMP was causally associated with increased estimated heel BMD (eBMD) (beta = 0.055; 95% CI: 0.020, 0.089; *P*_FDR_ = 0.001) and lumbar spine BMD (LS‐BMD) (beta = 0.103; 95% CI: 0.031, 0.175; *P*_FDR_ = 0.039). LMC was inversely associated with TB‐BMD in both discovery (beta = −0.207; 95% CI: −0.323, −0.091; *P*_FDR_ = 0.001) and replication cohorts (beta = −0.211; 95% CI: −0.335, −0.087; *P*_FDR_ = 0.002). LMP was negatively associated with TB‐BMD among individuals aged 15–30 but was positively associated in those aged 45–60. Additional MVMR analysis supported direct causal relationships of LMP and LMC with site‐specific BMD (eBMD and TB‐BMD) and age‐specific BMD (age 45–60).

**Conclusions:**

Our study observes the causal effects of LMP and LMC on BMD, with variations across anatomical sites and age groups. These findings enhance our understanding of the relationship between menstrual factors and bone health, providing evidence to inform targeted prevention strategies for OP.

## 1. Introduction

Osteoporosis (OP) is a systemic metabolic bone disorder characterized by reduced bone mass, deterioration of bone microarchitecture, and increased bone fragility. These factors collectively contribute to diminished quality of life, a higher risk of fractures, and disability [1]. Globally, the prevalence of OP is estimated at approximately 18.3%, affecting individuals across all ages and genders, with a notably higher disease burden in postmenopausal women [2, 3]. Bone mineral density (BMD) measurement remains the cornerstone for diagnosing osteopenia and OP [4]. Aging is accompanied by progressive declines in BMD and bone formation capacity, contributing to the global increase in OP prevalence [5]. Estrogen, a vital regulator of bone metabolism, is essential for preserving bone density [6]. Disruption of estrogen levels, such as those caused by late menarche or early menopause, can elevate the risk of bone loss and osteoporotic fractures [7].

Over the past two decades, a growing body of research has underscored the critical influence of menstrual factors on the occurrence and development of OP [8]. Previous studies have demonstrated that about one‐third of women reach their peak BMD within 4 years before or after menarche [9, 10]. Late age at menarche (AAM) and early age at menopause (last menstrual period [LMP]) have been linked to lower bone density and an elevated risk of osteoporotic fractures [11, 12]. Abnormal length of menstrual cycle (LMC), particularly when irregular or outside the typical 21‐ to 35‐day range, has been demonstrated to be associated with alterations in bone metabolism, formation, and remodeling [13, 14]. However, the findings of previous observational studies might be affected by several potential factors, including residual and reverse causation. Therefore, investigating the genetic causal pathways underlying the associations between menstrual factors and BMD/OP risk is essential.

Mendelian randomization (MR) is an epidemiological method that strengthens causal inference by utilizing genetic variants as instrumental variables (IVs) [15]. Due to the random distribution of genetic variants at conception according to Mendel’s assortment law of inheritance, MR is less prone to measurement errors and reverse causation than conventional observational studies [16]. Moreover, multivariable MR (MVMR) is a recently developed approach that simultaneously examines multiple independent but related exposures by integrating genetic variations of each risk factor into a single model [17].

In this study, we conducted a comprehensive MR analysis using site‐/age‐specific BMD to examine the causal relationship between menstrual factors (AAM, LMP, and LMC) and BMD. To identify the direct effect of menstrual factors on site‐/age‐specific BMD, additional MVMR analysis was conducted after adjusting for the effects of body mass index (BMI), polycystic ovary syndrome (PCOS), and smoking. This study offers novel insights into the impact of menstrual factors on BMD and may inform strategies for the management and prevention of OP.

## 2. Methods

### 2.1. Study Design

**Figure 1:**
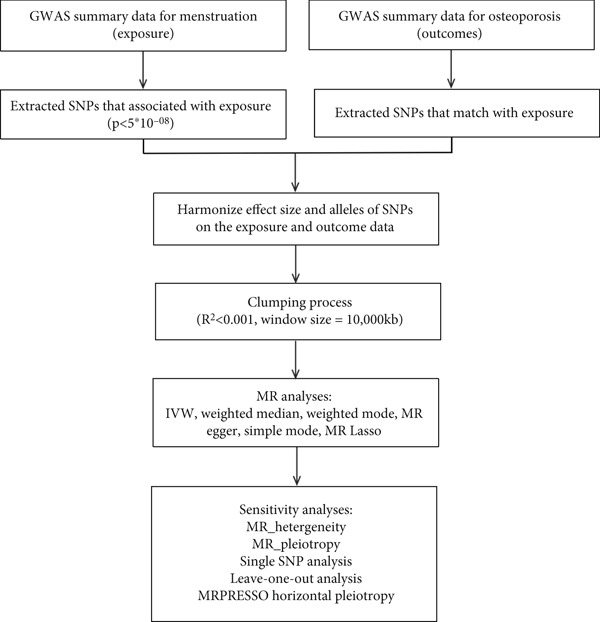
Schematic overview of the study design. GWAS: genome‐wide association study; SNPs: single‐nucleotide polymorphisms; IVW: inverse variance weighted; MR: Mendelian randomization.

This study employed a two‐sample MR analysis to investigate the causal associations between key menstrual factors (AAM, LMP, and LMC) and OP, as represented by site‐ and age‐specific BMD measures. In MR analysis, single‐nucleotide polymorphisms (SNPs) are commonly selected as IVs to estimate causal associations between exposures and outcomes. For these estimates to be valid, the following three critical assumptions must be met: (1) Genetic variants must exhibit a strong association with the exposure factors (association assumption), (2) genetic variants must be independent of confounders (independent assumption), and (3) genetic variants should affect the outcome exclusively through the exposure factors, meaning horizontal pleiotropy must be absent (excluding restrictive assumption) [18] (Figure 1).

All genetic data used in this study were obtained from the Genetic Factors for OP Consortium (GEFOS), and ethical approval was not required for these publicly available datasets. However, as the study primarily included individuals of European ancestry, the applicability of the findings to other populations may be limited.

### 2.2. Exposure Data Sources and IV Selection

We selected seven independent GWAS related to menstrual factors from the IEU OpenGWAS database (https://gwas.mrcieu.ac.uk/, accessed on October 1, 2024). For AAM (discovery), nine SNPs were identified from a GWAS with 29,346 participants (linkage disequilibrium [LD] < 0.001) [19]. In a GWAS study involving 143,819 subjects, 76 genetic variants for LMP (discovery) were identified (LD < 0.001), while seven genetic variants for LMC (discovery) were identified among 43,125 participants (LD < 0.002) (Table S1) [20]. In terms of replicate datasets for menstrual factors, there were 66 AAM‐related SNPs identified in a GWAS with 182,416 participants (LD < 0.001) [21], and five genetic variants for LMC (replicate) were obtained from a GWAS study involving 30,245 subjects (LD < 0.002) (Table S2) [22]. Notably, the LMC variable represents genetically influenced variation in average LMC derived from GWAS summary statistics. However, these data lack differentiation between regular and irregular cycles and do not provide categorical definitions of cycle length, which limits the granularity of the phenotypic assessment. Other key menstrual characteristics, such as total reproductive lifespan and amenorrhea history, were not included due to the absence of validated genetic instruments.

Genetic variants of menstrual factor exposure were identified from GWAS data of individuals of European descent. Subsequently, we selected SNPs associated with menstrual factors at genome‐wide significance *p* < 5 × 10^−8^ as instruments to obtain moderate IVs and then clumped these SNPs based on LD. Additionally, to mitigate the impact of weak IV bias on causal inference, we assessed the strength of each IV using the following formula: *F* = (*R* ^2^ × (*n* − *k* − 1))/(*k* × (1 − *R* ^2^)), where a *F*‐statistic greater than 10 indicates that the selected IVs are strong instruments [23].

### 2.3. Outcome Data Sources

When multiple GWASs were available for a single trait, we selected only the most recent and comprehensive study. Given that BMD is a reliable indicator for diagnosing OP, we used BMD as the outcome to represent the phenotype of OP. Five distinct site‐specific BMD measures were included: lumbar spine BMD (LS‐BMD), forearm BMD (FA‐BMD), femoral neck BMD (FN‐BMD), estimated heel BMD (eBMD), and total body BMD (TB‐BMD). To assess the causal impact of menstrual factors on TB‐BMD changes over the lifetime, TB‐BMD was further categorized by age groups: 0 < age ≤ 15, 15 < age ≤ 30, 30 < age ≤ 45, 45 < age ≤ 60, and age > 60 (Table S1). These age bands were defined based on the structure of the GWAS summary statistics and may not fully align with biological reproductive stages such as menarche or menopause.

The FN‐BMD, LS‐BMD, FA‐BMD, and TB‐BMD were assessed using dual‐energy X‐ray absorptiometry (DEXA), while eBMD was measured through quantitative ultrasound [24–26].

### 2.4. Statistical Analysis

In the univariable MR analysis, the inverse variance weighted (IVW) method was primarily used to investigate the causal relationship between genetic variants associated with the exposure and the outcome [25]. To assess the robustness of the results, sensitivity analyses were conducted using MR‐Egger, simple mode, weighted median (WM), weighted mode, and MR‐Lasso models.

The WM and MR‐Egger regression methods are also used to refine IVW estimates under more generalized conditions. The MR‐Lasso identifies and eliminates ineffective SNPs using Lasso regression by adding a penalty term to the causal effect estimator, which shrinks the regression coefficients toward 0, effectively forcing the coefficients of individual SNPs to equal 0 [26]. Additionally, the MR pleiotropy residual sum of squares and outlier (MR‐PRESSO) test was employed to identify potential horizontal pleiotropy and mitigate its effects by removing outliers [24]. Cochran’s *Q* statistic was used to assess heterogeneity, while a leave‐one‐out (LOO) sensitivity analysis was conducted to assess whether the observed findings were influenced by specific SNPs [27].

Previous studies have reported that BMI, smoking, and PCOS might be implicit factors for BMD and could infer the impact of menstrual factors on OP [28–31]. Therefore, we conducted an MVMR analysis to correct the influence of BMI, PCOS, and smoking and infer the direct effect of menstrual factors on BMD (Table S1).

Each exposure‐specific MR analysis was conducted independently, followed by a meta‐analysis to derive overall estimates of the effect of menstrual factors (both discovery and replicate datasets) on BMD. We used the *I* ^2^ statistic to assess heterogeneity together with the *p* value from Cochran’s *Q* test. When there was no heterogeneity, a fixed‐effect model meta‐analysis was applied to summarize the IV estimates from the two exposure databases for each exposure. In the presence of heterogeneity, a random‐effects model meta‐analysis was employed.

To control for multiple test problems (caused by multiple exposures), we used the Benjamini–Hochberg false discovery rate (FDR) method, setting the statistical significance threshold for MR effect estimates at *p* < 0.05 [32]. A *p* value of less than 0.05 was considered statistically significant. The statistical analyses were conducted using the R packages “Mendelian randomization,” “TwoSampleMR,” “meta,” “MVMR,” “ggplot2,” and “forest plot.”

## 3. Results

### 3.1. Causal Effects Between Menstrual Factors and Site‐Specific BMD

**Figure 2:**
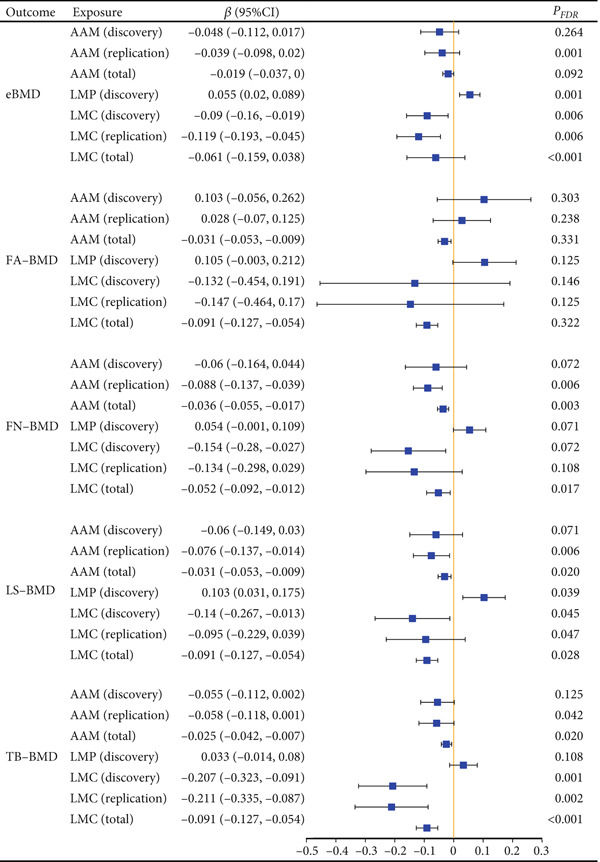
Causal association between menstrual factors and site‐specific BMD. BMD: bone mineral density; FA: forearm; FN: femoral neck; LS: lumbar spine; eBMD: estimated heel BMD; TB: total body; AAM: age at menarche; LMP: last menstrual period; LMC: length of menstrual cycle.

**Figure 3:**
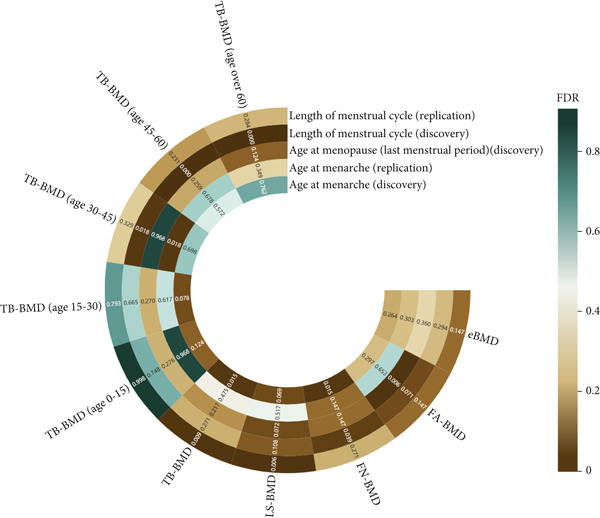
The results of FDR correction between menstrual factors and site‐/age‐specific BMD. BMD: bone mineral density; FA: forearm; FN: femoral neck; LS: lumbar spine; eBMD: estimated heel BMD; TB: total body.

Using the IVW method in univariate MR analysis, we found that LMP (discovery) was positively associated with both eBMD (beta = 0.055; 95% CI: 0.020, 0.089; *p* = 0.002; *P*_FDR_ = 0.001) and LS‐BMD (beta = 0.103; 95% CI: 0.031, 0.175; *p* = 0.005; *P*_FDR_ = 0.039) (Figures 2 and 3). In contrast, LMC exhibited a negative association with TB‐BMD in both the discovery LMC (beta = −0.207; 95% CI: −0.323, −0.091; *p* < 0.001; *P*_FDR_ = 0.001) and the replicate LMC cohort (beta = −0.211; 95% CI: −0.335, −0.087; *p* < 0.001; *P*_FDR_ = 0.002) (Figure 2 and Table S3). No other causal associations were observed between other menstrual factors and site‐specific BMD across datasets.

In the meta‐analysis, univariable MR estimates showed that LMC was negatively associated with eBMD (beta = −0.061; 95% CI: −0.159, 0.038; *p* < 0.001; *P*_FDR_ < 0.001), FN‐BMD (beta = −0.052; 95% CI: −0.092,−0.012; *p* = 0.004; *P*_FDR_ = 0.017), and TB‐BMD (beta = −0.091; 95% CI: −0.127, −0.054; *p* < 0.001; *P*_FDR_ < 0.001). Additionally, AAM was negatively associated with LS‐BMD (beta = −0.031; 95% CI: −0.053, −0.009; *p* = 0.006; *P*_FDR_ = 0.020), FN‐BMD (beta = −0.036; 95% CI: −0.055, −0.017; *p* < 0.001; *P*_FDR_ = 0.003), and TB‐BMD (beta = −0.025; 95% CI: −0.042, −0.007; *p* = 0.007; *P*_FDR_ = 0.020) (Figure 2).

These associations were further supported by alternative MR estimation methods, including MR‐Egger, WM, simple mode, weighted mode, and MR‐Lasso (Table S3). Cochran’s *Q* test generally indicated no significant heterogeneity, except in the association between menstrual factors and eBMD. MR‐Egger regression did not suggest substantial horizontal pleiotropy biasing the association between menstrual factors and BMD at specific sites (Table S4). Furthermore, LOO sensitivity analyses demonstrated that no single SNP drove the results (Figures S1, S2, S3, and S4).

### 3.2. Causal Effects Between Menstrual Factors and Age‐Specific BMD

**Figure 4:**
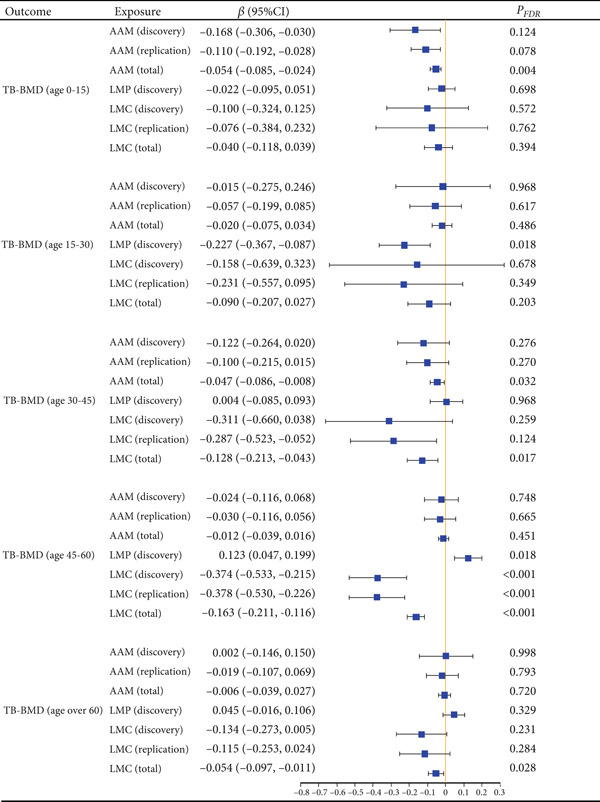
Causal association between menstrual factors and age‐specific BMD. BMD: bone mineral density; TB: total body; AAM: age at menarche; LMP: last menstrual period; LMC: length of menstrual cycle.

Univariable MR analysis using the IVW method unveiled that LMC was negatively associated with TB‐BMD among individuals aged 45–60 years, in both the discovery LMC cohort (beta = −0.374; 95% CI: −0.533, −0.215; *p* < 0.001; *P*_FDR_ < 0.001) and replicate LMC cohort (beta = −0.378; 95% CI: −0.530, −0.226; *p* < 0.001; *P*_FDR_ < 0.001) (Figures 3 and 4). In the discovery cohort, LMP was negatively associated with TB‐BMD in the 15–30‐year age group (beta = −0.227; 95% CI: −0.367, −0.087; *p* = 0.002; *P*_FDR_ = 0.018), but it was positively associated with TB‐BMD in the 45–60‐year age group (beta = 0.123; 95% CI: 0.047, 0.199; *p* = 0.002; *P*_FDR_ = 0.018) (Table S5). In the meta‐analysis, AAM was negatively associated with TB‐BMD in the 0–15 and 30–45 age groups. LMC was found to be negatively associated with TB‐BMD in individuals aged 30–45 years and those over 60 years (Figure 4).

These findings remained consistent across MR sensitivity methods, including MR‐Egger, WM, simple mode, and weighted mode (Table S5). The Cochran’s *Q* test indicated some heterogeneity (Table S6). The MR‐Egger regression generally did not reveal directional pleiotropy. Nevertheless, potential pleiotropy was observed for AAM in the discovery cohort and TB‐BMD among participants aged over 60 years (intercept *p* = 0.020) (Figure S5).

### 3.3. Direct Causal Effects of Single Menstrual Factors on Site‐/Age‐Specific BMD

To account for the possible effects of BMI, PCOS, and smoking (cigarettes smoked per day) on the relationship between LMP, LMC, and BMD, direct causal effects between each menstrual factor and site‐/age‐specific BMD were performed by MVMR analysis with the implementation of the IVW method after adjusting for the impacts of BMI, PCOS, and smoking. The outcomes of the MVMR revealed direct causal associations between LMP and LMC with both site‐specific BMD (eBMD and TB‐BMD) and BMD related to specific age groups (TB‐BMD age 45–60) (Table 1).

**Table 1:** Direct causal effect of menstrual factors on site‐/age‐specific BMD in MVMR analysis.

**Mediators adjusted**	**Effect of LMP (discovery) on eBMD**		**Effect of LMP (discovery) on LS-BMD**		**Effect of LMC (discovery) on TB-BMD**		**Effect of LMC (replicate) on TB-BMD**		**Effect of LMC (discovery) on TB-BMD (age 45–60)**		**Effect of LMC (replicate) on TB-BMD (age 45–60)**		**Effect of LMP (discovery) on TB-BMD (age 15–30)**		**Effect of LMP (discovery) on TB-BMD (age 45–60)**	
	**Beta (95% CI)**	**p**	**Beta (95% CI)**	**p**	**Beta (95% CI)**	**p**	**Beta (95% CI)**	**p**	**Beta (95% CI)**	**p**	**Beta (95% CI)**	**p**	**Beta (95% CI)**	**p**	**Beta (95% CI)**	**p**
BMI	0.064 (0.028, 0.099)	<0.001	0.061 (−0.014, 0.135)	0.109	−0.142 (−0.228, −0.056)	0.001	−0.190 (−0.317, 0.064)	0.003	−0.276 (−0.409, −0.143)	<0.001	−0.395 (−0.533, 0.257)	<0.001	−0.231 (−0.367, −0.095)	<0.001	0.106 (0.028, 0.185)	0.008
PCOS	0.064 (0.025, 0.102)	0.001	0.105 (0.023, 0.187)	0.012	−0.211 (−0.311, −0.091)	<0.001	−0.227 (−0.351, −0.103)	<0.001	−0.380 (−0.533, −0.228)	<0.001	−0.395 (−0.533, 0.257)	<0.001	−0.187 (−0.350, −0.024)	0.024	0.091 (0.007, 0.174)	0.034
Cigarettes smoked per day	0.056 (0.022, 0.090	0.001	0.085 (0.014, 0.156)	0.019	−0.199 (−0.357, −0.040)	0.014	−0.233 (−0.400, −0.065)	0.006	−0.362 (−0.546, −0.178)	<0.001	−0.409 (−0.595, 0.223)	<0.001	−0.221 (−0.365, 0.077)	0.003	0.103 (0.026, 0.181)	0.009

Abbreviations: BMD: bone mineral density; BMI: body mass index; eBMD: estimated heel BMD; LMC: length of menstrual cycle; LMP: last menstrual period (age at menopause); LS‐BMD: lumbar spine BMD; MVMR: multivariable Mendelian randomization; PCOS: polycystic ovary syndrome; TB‐BMD: total body BMD.

## 4. Discussion

Our study found that genetically determined menstrual factors of LMP and LMC were associated with site‐/age‐specific BMD, suggesting a causal effect between menstrual factors and risk of OP. Specifically, LMP was negatively associated with eBMD and LS‐BMD, indicating that the cessation of menstruation may contribute to a decrease in BMD at these sites. This reduction is likely due to a significant decline in estrogen levels during menopause, which impairs bone remodeling and repair processes, leading to bone loss and an increased OP risk [33]. Estrogen insufficiency is widely recognized as a key risk factor for OP [34]. Studies have demonstrated that low estrogen levels result in rapid and sustained bone loss [35]. Notably, weight‐bearing areas, such as the lumbar spine and femoral neck, are particularly susceptible to estrogen deficiency, which may explain the site‐specific nature of our findings [36].

We also observed a negative relationship between LMC and TB‐BMD, eBMD, and FN‐BMD, suggesting that irregular menstrual cycles are associated with site‐specific bone loss. Previous research has indicated that irregular or prolonged menstrual cycles, often exceeding the typical 21–35‐day range, may reflect chronic hypoestrogenism, which can compromise bone formation [37]. Furthermore, longer cycles may correlate with an extended follicular phase, during which estrogen levels remain reduced [38, 39]. The estrogen peak is delayed, and average estrogen levels are reduced throughout the cycle [40]. However, the lack of explicit categorization of cycle regularity or duration in our study may limit the interpretability of these results and may obscure specific hormonal patterns underlying BMD changes. Future research should comprehensively explore the cycle‐type–specific effects on bone health.

Among individuals aged 15–30, LMP was inversely associated with TB‐BMD, suggesting that younger women may be more likely to experience greater bone loss after the cessation of menstruation [41]. Parker et al. found that women who experienced menopause before the age of 50 had a higher risk of developing OP, and those with a menstrual history of less than 25 years had a significantly higher incidence of OP [42]. In contrast, in the 45–60 age group, LMP was positively associated with TB‐BMD, suggesting that BMD reduction slowed down or stabilized in women who experienced menopause during this period. A study performed by Svejme et al. demonstrated that for each additional year of postmenopausal age, the incidence of OP decreased by 4%, while the incidence of OP in postmenopausal women aged 50–54 decreased by 31% [43]. However, our broad age stratification may have limited interpretability, as the 15–30 age group includes both adolescents shortly after menarche and women in their reproductive prime, who likely exhibit distinct bone metabolism patterns. More refined age groupings (e.g., 12–18 and 19–30) might better capture the physiological transitions relevant to BMD variation.

Our study also showed that LMC is negatively correlated with TB‐BMD in the 45–60 age group, indicating that irregular menstrual cycles in middle‐aged and perimenopausal women may lead to bone loss. Such irregularities may disrupt bone metabolism and increase OP risk [37]. The outcomes of our meta‐analysis reinforced the negative correlation between AAM, LMC, and BMD at diverse skeletal sites across multiple age groups, underscoring the pivotal role of AAM and LMC in BMD dynamics [44].

Previous studies showed a causal link between menstrual factors and PCOS [45]. Menstrual factors may also play a role in women’s smoking‐related reactivity [46]. Chronic inflammation associated with PCOS and smoking is known to negatively affect bone health, and smoking increases the prevalence of OP [30, 31]. Notably, in our study, the MVMR analysis found that the effects of LMP and LMC on BMD were independent of known influencing factors such as PCOS and smoking. Although PCOS causes hormonal imbalances, direct evidence linking it to increased OP risk remains limited [47]. Another meta‐analysis showed that smoking had little or no effect on BMD in premenopausal women, regardless of its impact on estrogen levels [48]. In contrast, postmenopausal smokers experience faster bone loss but no reduction in plasma endogenous estrogen concentrations compared to nonsmokers [31]. Our study also found that BMI was involved in the causal association between LMP and LS‐BMD. BMI is an important factor affecting bone metabolism because body weight or obesity can affect bone density by changing hormone levels and increasing bone load [49]. In obese individuals, higher fat content may lead to elevated estrogen levels, which mitigate the adverse effects of menstrual factors on BMD [50]. Conversely, in individuals with lower body weight, reduced estrogen levels may elevate the risk of bone density loss [51]. Therefore, BMI may exacerbate or mitigate the effect of menstrual factors on BMD.

Although our MR and MVMR results support causal inferences, the absence of intermediate phenotypic markers, such as serum estrogen levels, follicle‐stimulating hormone (FSH) levels, or bone turnover markers (e.g., osteocalcin and *β*‐CTX), limits our ability to identify the biological pathways underlying these associations. These hormonal and inflammatory mediators may be critical in linking menstrual factors to skeletal metabolism. Future studies integrating genetic and phenotypic datasets are essential to bridging this gap.

Our study has several advantages. The extensive genetic data from European populations provided robust insights, reducing potential population structure bias, and the MR study design minimized confounding and reverse causation bias. In addition, the use of independent datasets and meta‐analytic techniques enhanced the statistical power and reliability of our findings. Furthermore, the site‐ and age‐specific BMD analyses offered valuable insights into the systemic impact of menstrual factors on bone health across various skeletal locations and age groups.

However, there are limitations to consider. First, as the GWAS data were derived primarily from individuals of European ancestry, this limits the applicability of our findings to other populations. The generalizability of these results to non‐European groups may be constrained, and future studies should aim to include more diverse populations to better understand the cross‐population relevance of these findings. Second, our exposure variables included only a partial aspect of menstrual history, specifically AAM, LMP, and LMC, without accounting for total reproductive lifespan, history of amenorrhea, or menopausal transition characteristics, all of which may influence bone outcomes. Third, MR analyses are inherently limited in that they cannot capture nongenetic variance and only account for a portion of the genetic effect. Therefore, the results should be interpreted with caution. Future research integrating comprehensive reproductive history, hormone levels, and biomarker data will be crucial to further elucidate these causal relationships.

## 5. Conclusions

Our study demonstrates the causal effects of genetic susceptibility to LMP and LMC on BMD reduction. Specifically, we provide evidence for a direct causal impact of LMC on age‐specific BMD in the 45–60 age group, highlighting the potential role of menstrual irregularities during perimenopause in contributing to bone loss in this population. However, the underlying biological mechanisms remain to be fully elucidated. These findings enhance our understanding of the relationship between menstrual patterns and bone health, offering valuable insights that could inform the development of targeted interventions and management strategies for the early prevention of OP.

## Ethics Statement

There is no need for ethical approval for the use of anonymous open data.

## Conflicts of Interest

The authors declare no conflicts of interest.

## Author Contributions

Sheng Li and Yan‐Yu Zhu conceived the study, searched the literature, and performed data extraction. Sheng Li conducted the primary statistical analysis, and Qian‐Qian Shi and Xiao Hu provided statistical expertise. Sheng Li and Yan‐Yu Zhu took the lead in writing the manuscript. Peng Wang and Hai‐Feng Pan contributed by critically revising the manuscript. Chun‐Rong Ma provided clinical expertise. Hai‐Fen Wei and Jia‐Jun Deng organized the charts and verified them. All authors provided critical feedback and helped shape the research, analysis, and manuscript. Sheng Li and Yan‐Yu Zhu are joint first authors:

## Funding

The study was funded by the National Natural Science Foundation of China, 10.13039/501100001809, 82404354 and 82273710; the Natural Science Foundation of Anhui Province, 10.13039/501100003995, 2108085Y26 and 2308085QH288; the Research Funds of Center for Big Data and Population Health of IHM, JKS2022017; the Key Scientific Research Foundation of the Education Department of the Province Anhui, 2022AH050653; and the Natural Science Foundation of Anhui Medical University, 2022xkj006.

## Supporting Information

Additional supporting information can be found online in the Supporting Information section.

## Supporting information


**Supporting Information 1** Figure S1 Sensitivity analyses for the causal effects of LMC (replicate) on eBMD. (A) Scatter plot, (B) funnel plot, (C) forest plot, and (D) LOO plot. BMD: bone mineral density; eBMD: estimated heel BMD; LMC: length of menstrual cycle.

**Supporting Information 2** Figure S2 Sensitivity analyses for the causal effects of AAM (discovery) on FN‐BMD. (A) Scatter plot, (B) funnel plot, (C) forest plot, and (D) LOO plot. BMD: bone mineral density; AAM: age at menarche; FN: femoral neck.

**Supporting Information 3** Figure S3 Sensitivity analyses for the causal effects of LMC (discovery) on FN‐BMD. (A) Scatter plot, (B) funnel plot, (C) forest plot, and (D) LOO plot. BMD: bone mineral density; LMC: length of menstrual cycle; FN: femoral neck.

**Supporting Information 4** Figure S4 Sensitivity analyses for the causal effects of LMC (discovery) on LS‐BMD. (A) scatter plot, (B) funnel plot, (C) forest plot, and (D) LOO plot. BMD: bone mineral density; LMC: length of menstrual cycle; LS: lumbar spine.

**Supporting Information 5** Figure S5 Sensitivity analyses for the causal effects of AAM (replicate) on TB‐BMD (age 0–15). (A) Scatter plot, (B) funnel plot, (C) forest plot, and (D) LOO plot. BMD: bone mineral density; AAM: age at menarche; TB: total body.

**Supporting Information 6** Supporting information tables.

## Data Availability

Data available on request from the authors.
