# Supplementary figures and images for: Genetic Insights Into the Relationship Between Menstrual Factors and Site‐/Age‐Specific Bone Mineral Density
Source: Int J Genomics. 2025 Nov 10;2025:9979878. doi: 10.1155/ijog/9979878 (PMC12598576; doi:10.1155/ijog/9979878)

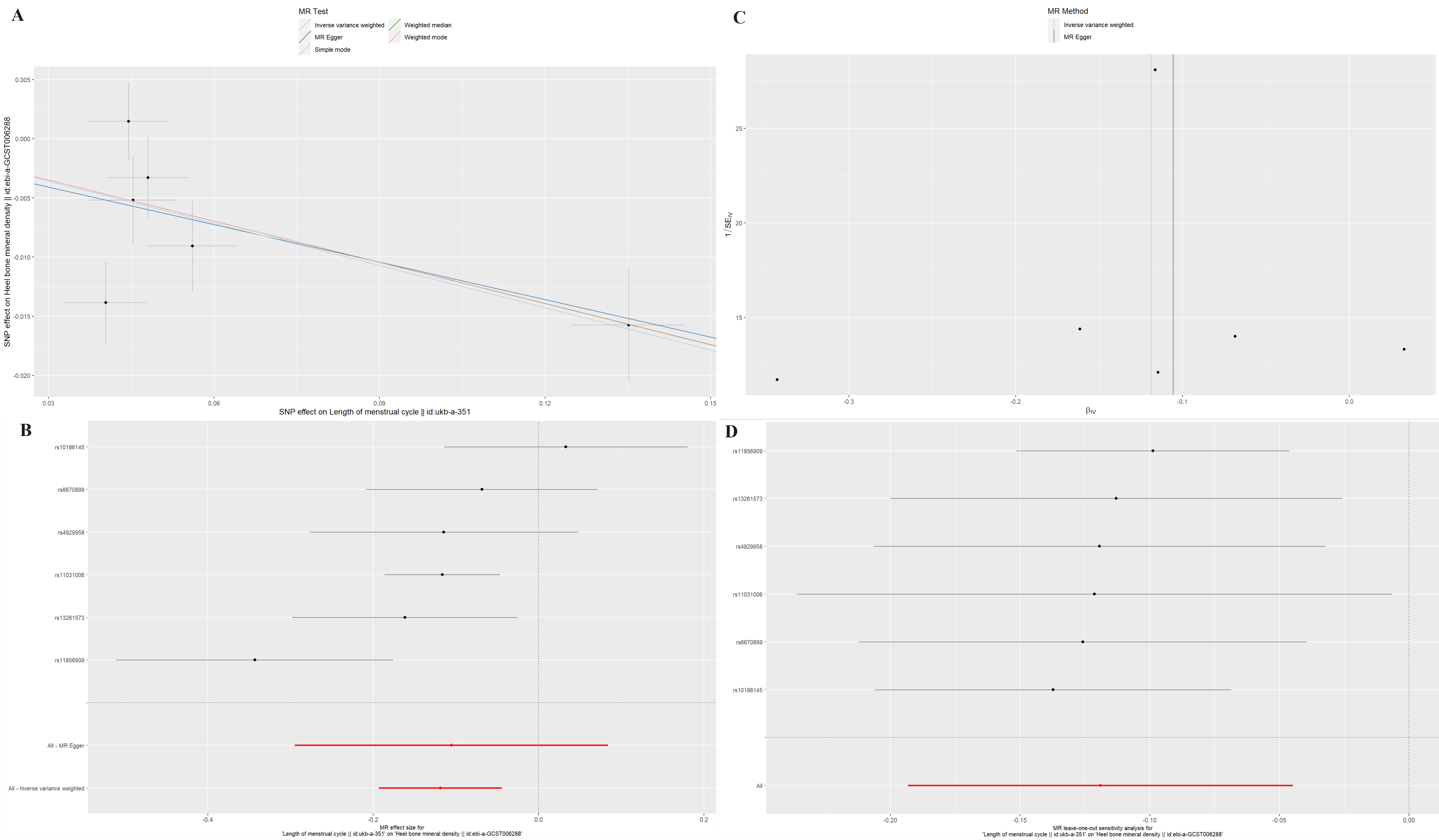

Supplement: Supplementary file 1 — Supporting Information 1 Figure S1 Sensitivity analyses for the causal effects of LMC (replicate) on eBMD. (A) Scatter plot, (B) funnel plot, (C) forest plot, and (D) LOO plot. BMD: bone mineral density; eBMD: estimated heel BMD; LMC: length of menstrual cycle. [file IJOG-2025-9979878-s001.tif]

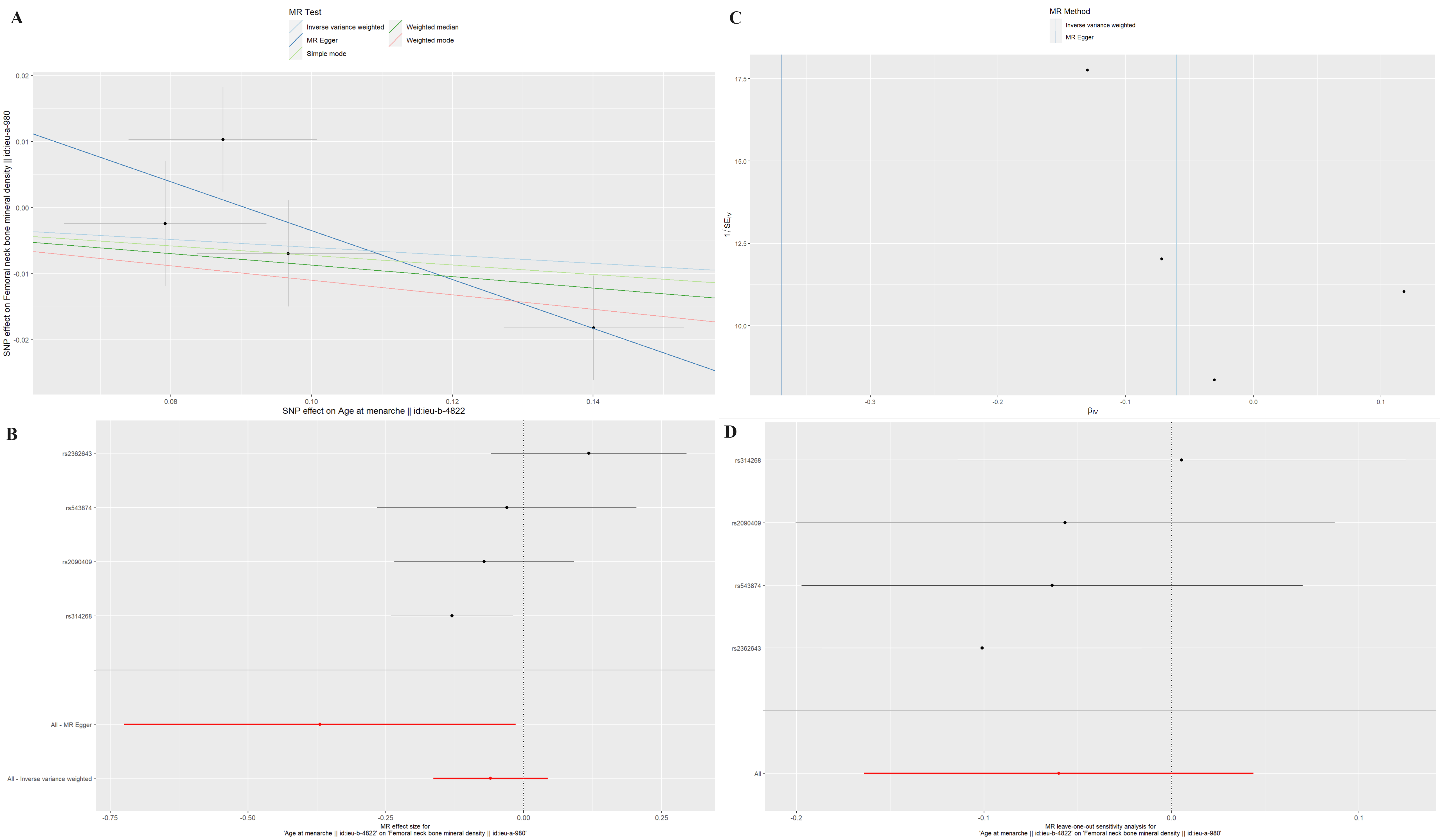

Supplement: Supplementary file 2 — Supporting Information 2 Figure S2 Sensitivity analyses for the causal effects of AAM (discovery) on FN‐BMD. (A) Scatter plot, (B) funnel plot, (C) forest plot, and (D) LOO plot. BMD: bone mineral density; AAM: age at menarche; FN: femoral neck. [file IJOG-2025-9979878-s002.tif]

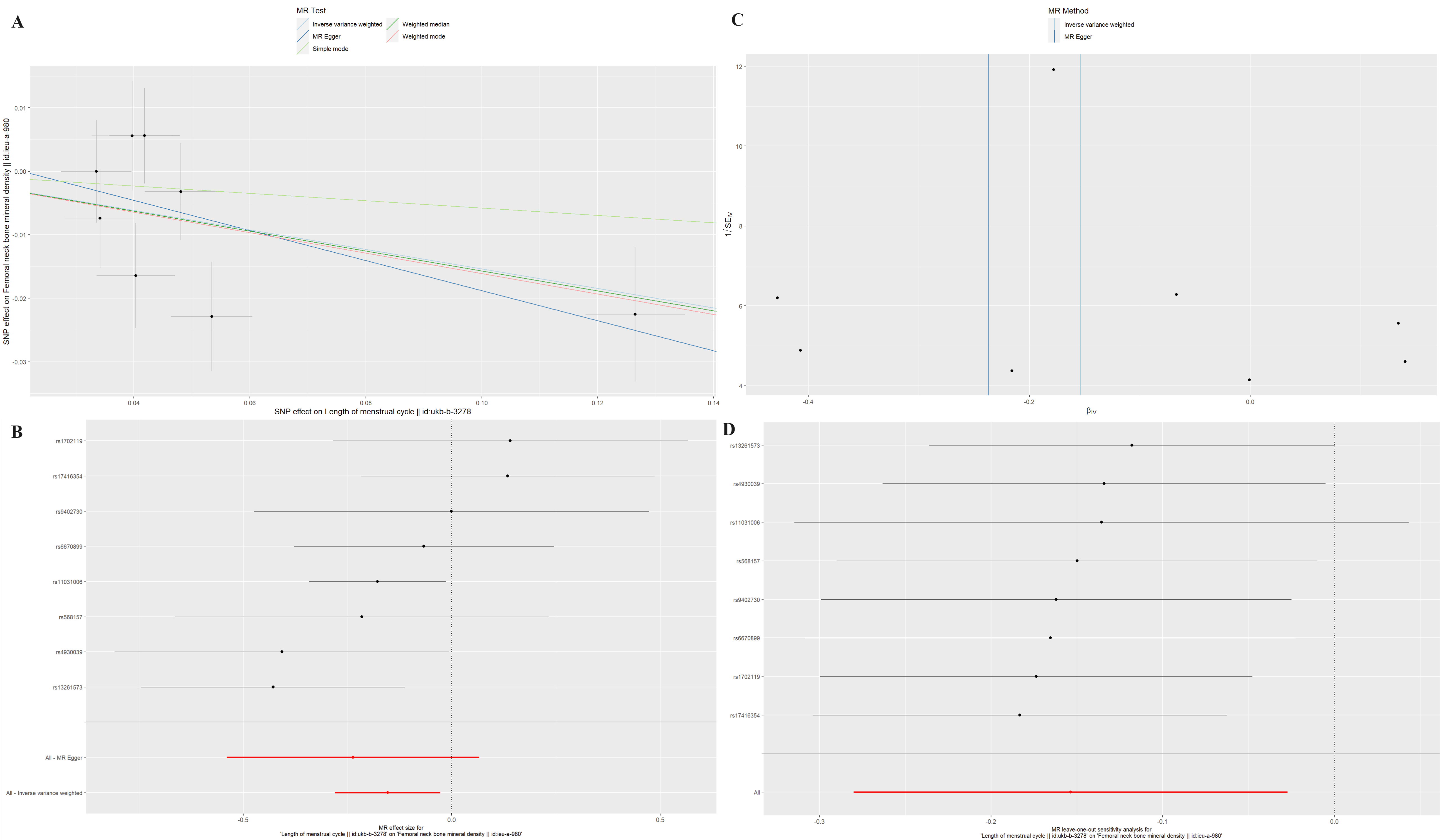

Supplement: Supplementary file 3 — Supporting Information 3 Figure S3 Sensitivity analyses for the causal effects of LMC (discovery) on FN‐BMD. (A) Scatter plot, (B) funnel plot, (C) forest plot, and (D) LOO plot. BMD: bone mineral density; LMC: length of menstrual cycle; FN: femoral neck. [file IJOG-2025-9979878-s004.tif]

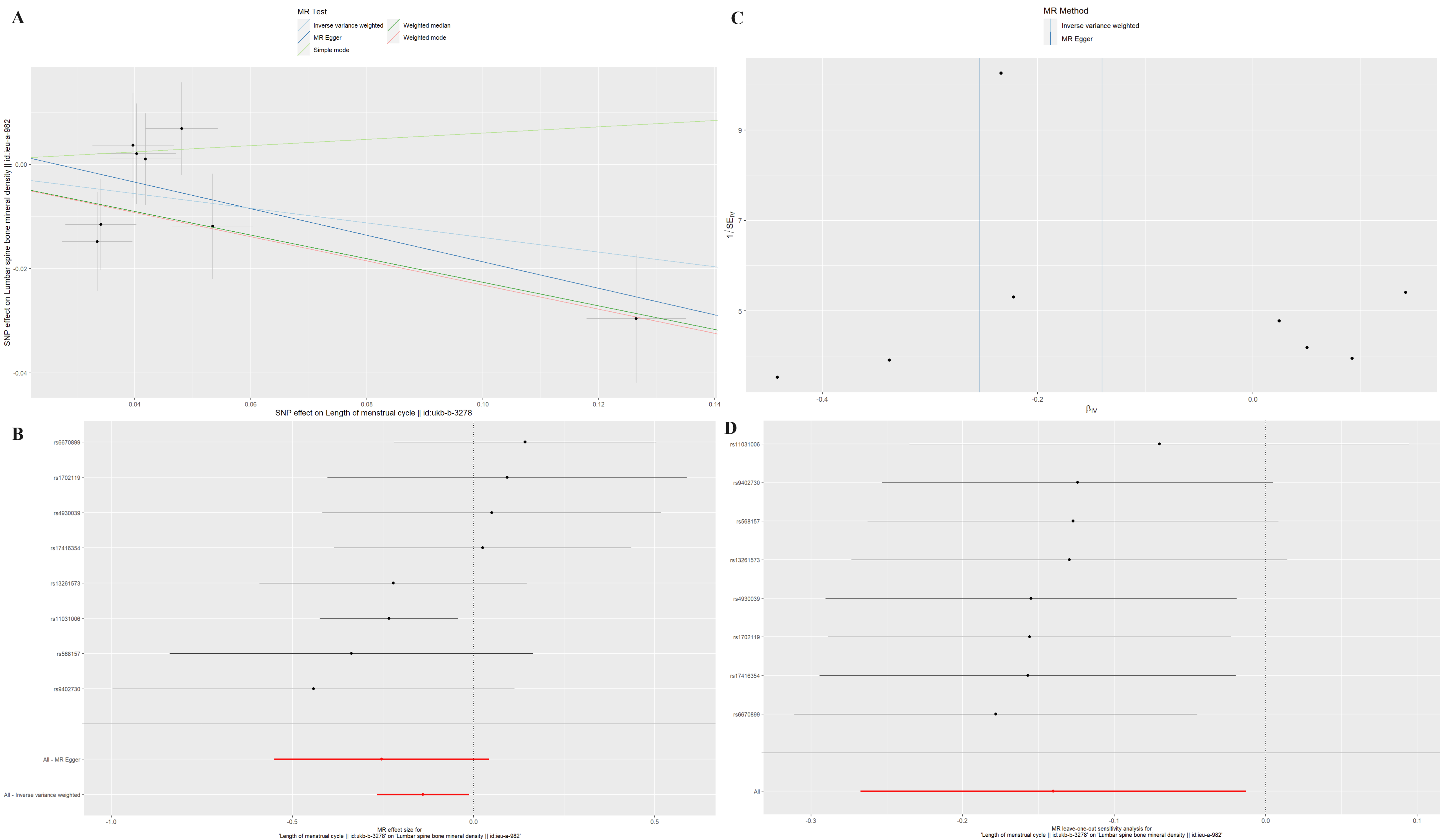

Supplement: Supplementary file 4 — Supporting Information 4 Figure S4 Sensitivity analyses for the causal effects of LMC (discovery) on LS‐BMD. (A) scatter plot, (B) funnel plot, (C) forest plot, and (D) LOO plot. BMD: bone mineral density; LMC: length of menstrual cycle; LS: lumbar spine. [file IJOG-2025-9979878-s003.tif]

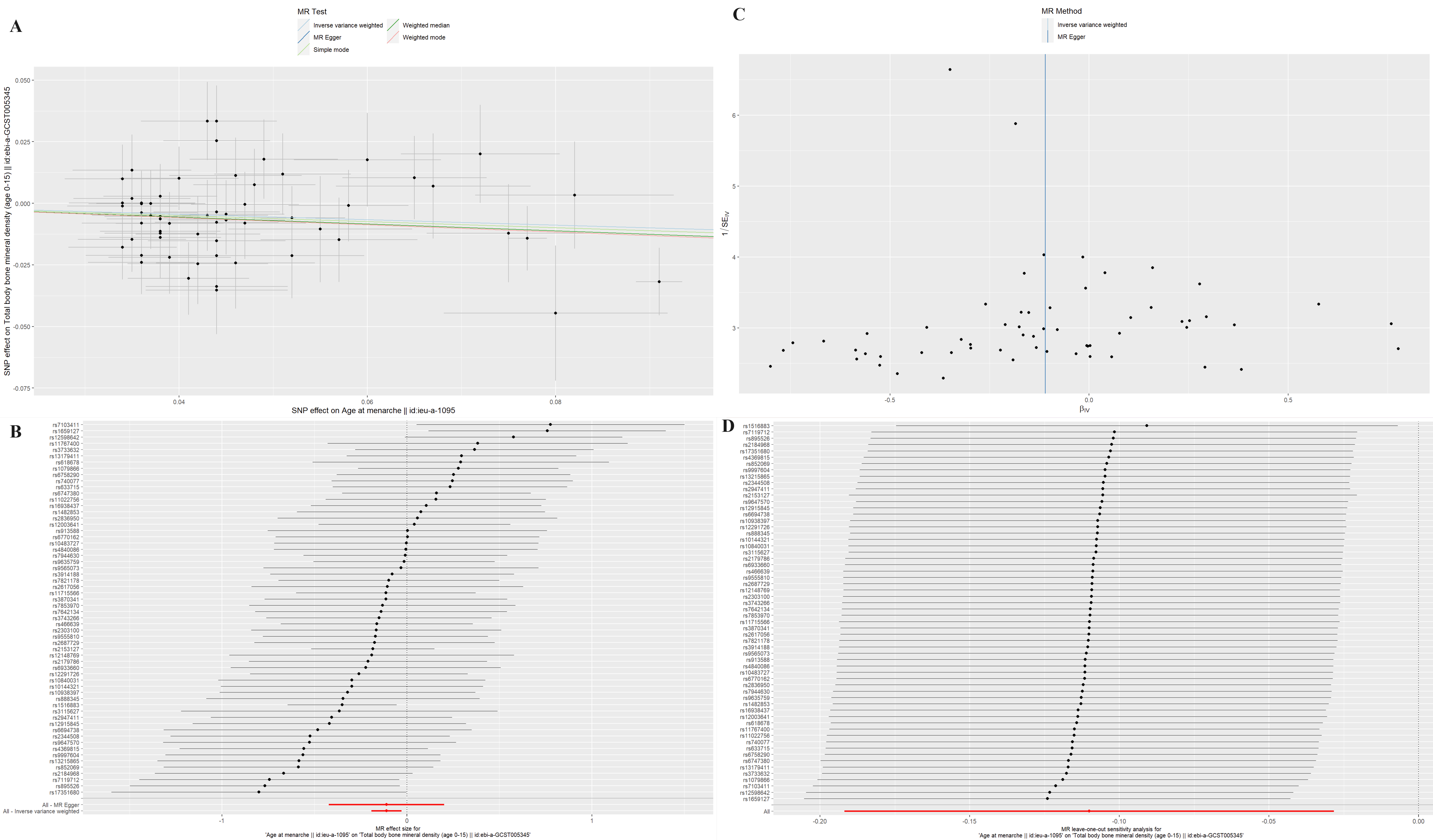

Supplement: Supplementary file 5 — Supporting Information 5 Figure S5 Sensitivity analyses for the causal effects of AAM (replicate) on TB‐BMD (age 0–15). (A) Scatter plot, (B) funnel plot, (C) forest plot, and (D) LOO plot. BMD: bone mineral density; AAM: age at menarche; TB: total body. [file IJOG-2025-9979878-s006.tif]
